# Politics in Dental Associations

**Published:** 1895-02

**Authors:** 


### Editorial.

### POLITICS IN DENTAL ASSOCIATIONS.

It will, perhaps, ever remain a difficult matter to arrange professional societies without more or less friction. As long as human nature remains as it is, with its varied and oftentimes discordant elements, it will be difficult, if not impossible, to harmonize antagonistic forces and place these bodies independent of political influence. It must be conceded that inasmuch as professions are, or should be, based on altruistic motives of action, seeking the greatest good possible to suffering humanity, they should eliminate all selfish considerations.

Unfortunately, while this is the true basis of all professional work, whether in the individual or in a collective capacity, there is an ever-increasing tendency to use the good for selfish ends.

Dental organizations, following the custom of the medical and other societies, have generally based their organic laws upon a principle that invites, sooner or later, to caucus work, combining for the overthrow of those in power, that another clique may gain supremacy.

While the members of an organization remain in harmonious relations, the old form of constitution, with its list of president, vice-president, secretary, etc., answers an excellent purpose; but it has been abundantly demonstrated, in and out of professional work, that this is but temporary, and in the course of time personal aspirations for the highest offices, or, what may be considered worse, the desire to overthrow the ruling body, brings about a conflict, and the organization is given over to political interests and political methods, which eventually injures the usefulness of the society and possibly ends in its destruction.

The American Dental Association was a sufferer for years from this infliction, and in degree is still weakened by it. The presidency was to many more important than the work the members met to accomplish, and long anterior to the annual gathering more attention was paid by opposing forces to securing the coveted prize than to scientific work needed to make the meeting of permanent value. Who of the members cannot recall the scenes that annually occurred at these gatherings? So much was this resorted to that a majority of the members finally became convinced that this evil influence must be destroyed root and branch, and a committee was appointed to prepare a constitution that would make politics an impossibility. When this new constitution becomes operative it is hoped that the American Dental Association will have heard the last of political cabals, and that members will meet together for purely professional work.

While the future promises well for this organization, its previous example has worked a serious injury to related and subordinate bodies. We have not the constitutions of the various societies by us for reference, but it is presumed very few have anything in their organic laws that antagonizes this baleful influence. From the reports that come to us, here and there, of societies broken up, or, to use the political word, "captured," it is inferred that most of those composing these bodies have not yet reached the advanced position taken by medical men on this question.

Very recently the College of Physicians in Philadelphia was the scene of a similar effort of a very mild character compared to that frequently seen in dental organizations. Officers were to be elected, and one over-zealous individual wrote a letter to various Fellows advocating the election of a prominent member. The tactics of the gentleman were exposed, and the candidate was defeated, and a strong probability is entertained that the result will be the expulsion of the member who sought to attain his end by political measures. This is stated simply to show that the professional feeling in medical circles is positively antagonistic to any such methods. There can be no reason why the same feeling should not be maintained in the dental profession.

There is only one way, it seems to the writer, to meet this deep-seated poison in the professional body, and that is to make an entire change in the organic laws. One society, recently organized, has taken the election of officers almost entirely out of the hands of the main body. The members have the privilege of nominating a certain number for each office, and from these the council must select one. It is hoped by this method, similar, we believe, to that in force in the National Medical Association, that the disturbing element of politics will be entirely eradicated. There are some objections to this mode of doing business, as its tendency is to a centralization of power and a partial deprivation of the individual right of expression, so dear to the average American; but some sacrifices must be made for the good of the whole, and this plan has less of evil in it than that ordinarily adopted.

If we can rid our organizations of this political tendency, we may attempt other reforms; but while men who are working solely for the advancement of the profession, and care nothing for so-called posts of honor, are constantly to be confronted with political warfare, they will lose interest and decline to take an active part. It is for this reason, it is surmised, that some of the best men in dentistry rarely or never are seen at the annual gatherings, preferring to do their work in their own local organizations. A total change must be made in this direction, or the result will be that new organizations will spring up in which only those can enter who are willing to lay aside all selfish aspirations and work solely for the true advancement of the associations in scientific knowledge, and not for the ephemeral glorification of the individual.

